# Differences in time–intensity sensory profiles of sweet taste intensity of glucose between older and young adults

**DOI:** 10.3389/fnut.2024.1273055

**Published:** 2024-03-28

**Authors:** Hirotaka Wada, Hideki Matsumoto, Mutsumi Takagiwa, Hitomi Sato, Kyoko Ishiguchi, Aya Inoue, Tazuko K. Goto

**Affiliations:** ^1^Department of Oral and Maxillofacial Radiology, Tokyo Dental College, Chiyoda-ku, Tokyo, Japan; ^2^Laboratory of Mathematics, Tokyo Dental College, Chiyoda-ku, Tokyo, Japan; ^3^Tokyo Dental College Research Branding Project, Tokyo Dental College, Chiyoda-ku, Tokyo, Japan; ^4^Faculty of Dentistry, The University of Hong Kong, Sai Ying Pun, Hong Kong SAR, China

**Keywords:** older adult, sweet taste, glucose, time–intensity, humans

## Abstract

**Background:**

To understand age-related changes in sweet taste perception in daily life, it is important to understand taste intensity at the suprathreshold level. Previous studies have attempted to characterize the temporal aspects of human taste perception in terms of time–intensity evaluations. The perception of dynamic taste intensity in older adults increases slowly for salty taste; however, there have been no previous studies on time–intensity sensory evaluation of sweet taste in older adults. We hypothesized that older adults perceive sweet taste intensity more slowly than young adults.

**Methods:**

Fifty young and 40 older adults participated in the study and glucose solutions of 0.6 M and 1.5 M were used as stimuli. The study comprised two experiments: (1) a cup tasting test (static taste perception in the mouth), and (2) a time–intensity sensory evaluation, in which the solutions were presented using a custom-made delivery system. The intra-oral device was made to fit each participant’s dentition. Further, the level of gag reflex was taken into consideration for each participant in the design of the intra-oral device. A suction tube was placed across the posterior tongue near the throat to remove solution and saliva. The solution delivery system was controlled by an original computer program.

**Results:**

Older adults presented significantly different maximum intensity timing and slope for both concentrations compared with young adults (slope for 1.5 M, *p* < 0.01; others, *p* < 0.05). No significant differences were found between the older and young adults for reaction timing and maximum intensity.

**Conclusion:**

We conclude that older adults perceived sweetness more slowly than young adults, and ultimately perceived almost the same intensity as young adults. This is the first reported characterization of the time–intensity profile of sweet taste intensity of glucose in older adults. Using a standardized system enabled us to assess and compare feedback on taste intensities among different age groups in real-time. Based on this, we recommend older adults “savor” to perceive sweet tastes at the same level experienced by young adults.

## Introduction

Glucose is the primary fuel for life and cellular uptake of glucose is a fundamental process for metabolism, growth, and homeostasis (1). The adult human brain generally represents about 2% of total body mass but consumes approximately 25% of the glucose supply (1). The cognitive requirement for glucose may not change markedly with aging and older adults should consume an adequate amount of glucose daily. However, the intake of too much sugar increases the risk of obesity, diabetes, and dental caries (2–4). The World Health Organization (WHO) recommends reducing the intake of free sugars to less than 10% of total energy intake (5). To understand age-related changes in static taste perception in humans, the detection threshold (the minimum concentration at which a participant can reliably distinguish between water and taste) (6–9), the recognition threshold (the minimum concentration at which a participant can distinguish taste quality, such as sweet or salty) (10–18) and the suprathreshold (the stimulus that is large enough to produce a detectable physiological effect) have been studied (19–28). Studies using the whole-mouth method (11–16) and the filter paper disc method (17) have reported that sweet taste detection thresholds and recognition thresholds increase with age. However, three studies of sweet taste at the suprathreshold level report that the taste intensity of sweet solutions is lower in older adults than in young adults (19–21), while six studies found no significant difference (22–27).

Previous studies have attempted to characterize the temporal aspects of taste perception in young adults, in terms of time–intensity evaluations (29–36). However, there have been no previous studies of time–intensity sensory evaluation of taste in older adults except for a study of salty taste, which showed that the perception of dynamic taste intensity in older adults increases slowly (37). Hence, we hypothesized that older adults perceive sweet taste intensity more slowly than young adults.

## Materials and methods

### Participants

Ninety healthy adults were recruited for the study. The older adult group consisted of 40 individuals (20 men, 20 women), mean and standard deviation (SD) were 70.1 ± 6.7 in 60–85 years and body mass index (BMI), 23.0 ± 2.5 kg/m^2^. The young adult group comprised 50 individuals [25 men, 25 women; age, 21–34 years (26.7 ± 3.3); BMI, 21.7 ± 3.0 kg/m^2^] (Table 1). This study included 16 older adults and 8 young adults included in a previous study (37). This study was a preliminary study, therefore, we set the number of participants per group tentatively. For reference, the number of participants in previous sweet taste studies was 14–80 participants in cup tasting tests and filter paper disk tests (6–27), and 7–20 participants in time–intensity sensory evaluations (29–36). Advertisements around Tokyo Dental College were used to recruit participants between April 2019 and March 2022. Individuals who met at least one of the following criteria were excluded from the study: (1) smokers, (2) reported to have difficulties using the intensity meter during time–intensity recording, (3) older adults with Mini-Mental State Examination scores of 22 or less (38) and suspected dementia, and (4) people with taste, smell, psychiatric, or neurological disorders. Ten older adults had hypertension, reflux esophagitis, hyperlipidemia, and rheumatoid arthritis and took medication. The information sheets for these medications listed taste disorder as a side effect at the following incidence rates: at less than 0.1% for one medication, 0.1%–5% for one medication, less than 0.3% for one medication, 0.5%–1% for one medication, less than 1% for one medication, and unknown for six medications. However, participants on medication were not largely affected by their medication and they could distinguish the two glucose solutions of different concentrations used in this study. No enrolled participants were excluded from our study.

**TABLE 1:** Demographic data.

	Older	Young
Number of participants	40	50
Age, mean (SD)	70.1 (6.7)	26.7 (3.3)
Sex (male/female)	20/20	25/25
BMI, mean (SD)	23.0 (2.5)	21.7 (3.0)

SD, standard deviation; BMI, body mass index.

The participants were ordinary citizens and not specially trained. The study was conducted according to the Ethical Guidelines for Medical and Health Research Involving Human Subjects of the Ministry of Health, Labour and Welfare and the Ministry of Education, Culture, Sports, Science, and Technology, Japan and the Declaration of Helsinki on Biomedical Studies Involving Human Subjects (39). The study was approved by the Institutional Review Board of Tokyo Dental College (No. 676), and all participants provided written informed consent.

We assigned registration numbers to the data. The data were collected between April 2019 and March 2022. Correspondence tables between registration numbers and data were strictly managed. The data are not identifiable to anyone other than the registered researchers.

### Taste solutions

The 0.6 M (108 g/L) and 1.5 M (270 g/L) glucose solutions were prepared with distilled water. Based on the results of a pilot study, we chose 0.6 M and 1.5 M glucose because participants could differentiate between the two concentrations (0.5 M, 0.7 M, and 1.0 M concentrations were not chosen), and they could be completely rinsed from the tongue. The 0.6 M and 1.5 M concentrations were based on estimations for soft drink and honey, respectively. We did not disclose the composition or intensity of the taste solutions to the participants.

Distilled water was used to wash out the glucose solutions and also as the control. All solutions were kept at 25°C.

### Experimental design

This study comprised two experiments.

Experiment 1: cup tasting (static taste perception in the mouth).

Experiment 2: time–intensity sensory evaluation (dynamic taste perception in the mouth).

Experiments 1 and 2 were conducted on the same day for a maximum of three participants because of the many processes involved in the experiment and the time restriction of the hospital.

### Experiment 1: cup tasting

**FIGURE 1:**
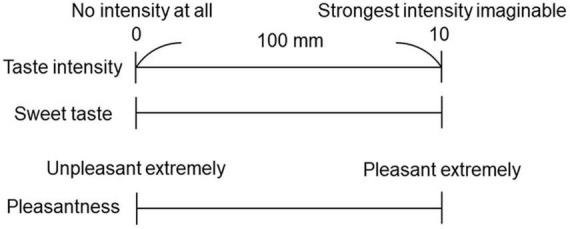
Visual analog scale (VAS) for the questionnaire. A 100 mm horizontal straight line was used to represent the VAS. Participants rated the taste intensity of the overall taste, sweet taste intensity, and pleasantness.

Each participant performed the cup tasting test once for each of the solutions (0.6 M and 1.5 M). Six milliliters of taste solution was sipped from an unlabeled paper cup, held in the mouth without gargling or swallowing, and spat out after 6 s. The mouth was then washed with distilled water, and the taste rating recorded on a paper sheet. Participants rated the intensity of the overall taste (sum of all taste qualities perceived), sweet taste intensity, and the pleasantness of the solution on a 0–10 visual analog scale (VAS); “0” represented “no intensity at all” and “10” represented the “strongest intensity imaginable” (Figure 1). This method with no gargling or swallowing was consistent with experiment 2, in which the solution flowed only over the tongue. Participants were provided distilled water in another paper cup to completely rinse the residual taste before receiving another solution.

### Experiment 2: time–intensity sensory evaluation

**FIGURE 2:**
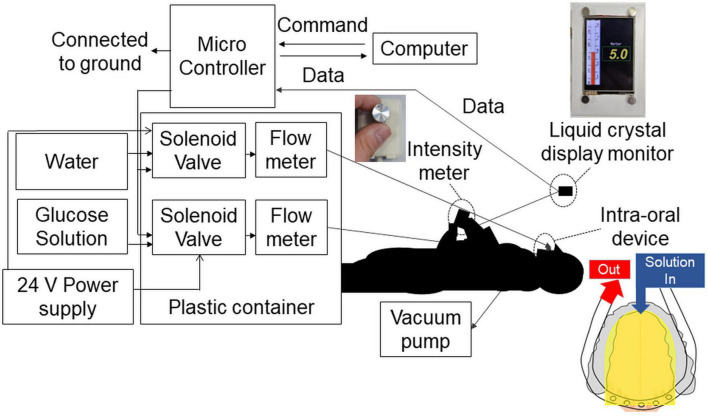
Time–intensity sensory evaluation meter and solution delivery system. The combined time–intensity sensory evaluation meter and computer-controlled taste stimulus delivery system were synchronized. The intra-oral device was made to fit each participant’s dentition. Furthermore, for each participant, the level of gag reflex was taken into consideration in the design of the intra-oral device. The solution flowed to the dorsal and lateral sides of the tongue covering the fungiform and anterior half of the foliate papillae (yellow areas in the figure). The solution and saliva were removed by a suction tube placed at the back of the mouth. The overall perceived taste intensity was recorded by participants using the rotary dial on a hand-held time–intensity sensory evaluation meter during administration of the taste solution. The scale displayed on a liquid crystal display monitor corresponded to the taste intensity, ranging from 0 (no taste) to 10 (strongest taste imaginable).

For time–intensity sensory evaluation, the solution was presented to each participant’s tongue using the taste solution delivery system under standardized conditions (40) (Figure 2). The intra-oral device was made to fit each participant’s dentition. Ten days before the experiment, dentists took an impression of each participant’s dental arch. The impression was disinfected with 0.1% sodium hypochlorite with standard precautions to prevent infection, and a working model was made. This process took 90 min. We then designed and fabricated an intra-oral device for each participant, with consideration of the level of gag reflex (40), which took 10 days.

The solution was supplied to the lateral and dorsal sides of the tongue and adjusted to ensure coverage of the fungiform papillae and the anterior half of the foliate papillae. A suction tube was placed across the posterior tongue near the throat to remove solution and saliva so that the participants did not need to swallow them.

The solution delivery system was controlled by an original computer program. The flow rate of solutions was constant at 110 ml/min and the participant did not feel any tactile sensations (40).

A prior study by Gotow et al. (41) found that when measuring time–intensity curves of bitterness, the perceived intensity was lower in the initial trial when compared with the second, third, and fourth trials. Additionally, the perceived intensity-time course did not differ among the second, third and fourth trials (41). Gotow et al. (41) suggested that untrained participants need a training trial using a warm-up sample before starting the test to obtain reliable performance in time–intensity evaluation (41). Therefore, at the beginning of experiment 2 we delivered distilled water onto the tongue of each participant for approximately 5 min to check the system. Warm-up samples (taste solution and water) were then each delivered once to each participant’s tongue. Formal testing then commenced as described below.

**FIGURE 3:**
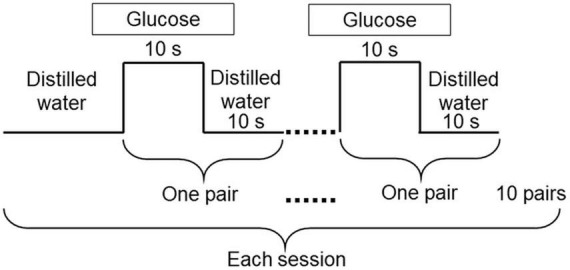
Experimental design. The experiment was carried out using 0.6 M and 1.5 M glucose solutions. Each session started with distilled water, followed by 10 pairs of glucose for 10 s, and distilled water for 10 s. Distilled water was used to wash out the glucose solutions. Only one concentration was used within each session.

A block design was employed. The experiment started with the 0.6 M glucose solution (the lower concentration solution) and we then delivered the 1.5 M solution by the same method. Only one concentration within one session was used (37). A session consists of 10 pairs of glucose and distilled water delivery for 10 s each (Figure 3). The 10 s stimulus duration was chosen based on studies showing that participants perceive maximum intensity of sucrose solutions between 5.0 and 10 s (29), and 6.0 s (36). The 10 s stimulation was therefore long enough to examine maximum intensity timing for 0.6 M and 1.5 M solutions. Complex methods were avoided so that older adults could concentrate on assessing taste intensity without being fatigued. We also recorded a decline in perceived sweet taste intensity during washing out. These data are not reported in this paper because the disappearance of the sweet sensation was not physiological but artificial (Figure 3) (37).

The overall perceived taste intensity was recorded by participants using the rotary dial developed by Goto et al. (40) (Figure 2). The intraoral device and solution delivery system was also designed by Goto et al. (40). The rotary dial consisted of a variable resistor and a 13 mm diameter knob. The scale on the dial corresponded to a taste intensity, ranging from 0 (no taste) to 10 (strongest taste imaginable) (37, 40). A computer-controlled taste solution delivery system connected to the intra-oral device was synchronized with the time–intensity evaluation meter, and participants’ perceptions were monitored in real-time (37, 40). In this study, we modified the system and the values recorded by the dial were displayed on a digital monitor. Older adults were less fatigued by this new design. An original computer program was written for this study.

The experimental conditions were fixed. An author (H.W.) communicated with the participants to obtain informed consent and to provide instructions for the experiments with the aid of an explanatory leaflet. Participants attended the experiments in the morning and in the same room. The room temperature was 24.0 ± 1.0°C.

### Questionnaire evaluation after time–intensity sensory evaluation

Immediately after the time–intensity sensory evaluations, participants rated pleasantness, sweet taste intensity, and taste intensity (Figure 1).

### Data analysis and statistics

We used R software 3.4.1^1^ for statistical analyses. We set statistical significance at *p* < 0.05.

### Taste solutions

A Wilcoxon signed-rank test was performed on the nonparametric and paired data to measure the reported differences in sweet taste between 0.6 M and 1.5 M glucose solutions. We used this test in the cup tasting test and in the time–intensity sensory evaluation.

### Questionnaires

We used the Shapiro–Wilk test to investigate the normality of the data distribution. As a result, a nonparametric test was applied; the Mann–Whitney U test was used to test for significant differences between older adults and young adults in taste intensity, sweet intensity, and pleasantness. Multiple comparisons were not performed because two groups were compared according to the hypotheses.

### Time–intensity sensory evaluation

The time–intensity profiles were analyzed with MATLAB R 2019a (The MathWorks, Inc., Natick, MA, USA). We carefully observed all profiles and included nearly all of the data. Strange profiles caused by the following operational errors were excluded from the study: (i) never turned the rotary dial during taste solution delivery, (ii) did not turn the rotary dial during taste solution delivery but did turn the rotary dial during water delivery, (iii) turned the dial more when water came onto the tongue. As a result, 1.4% (0.6 M young adults), 1.4% (1.5 M young adults), 4.3% (0.6 M older adults), and 3.0% (1.5 M older adults) of the profiles were excluded. Then, the average of all profiles and the standard error of the mean (SEM) were calculated for each condition.

**FIGURE 4:**
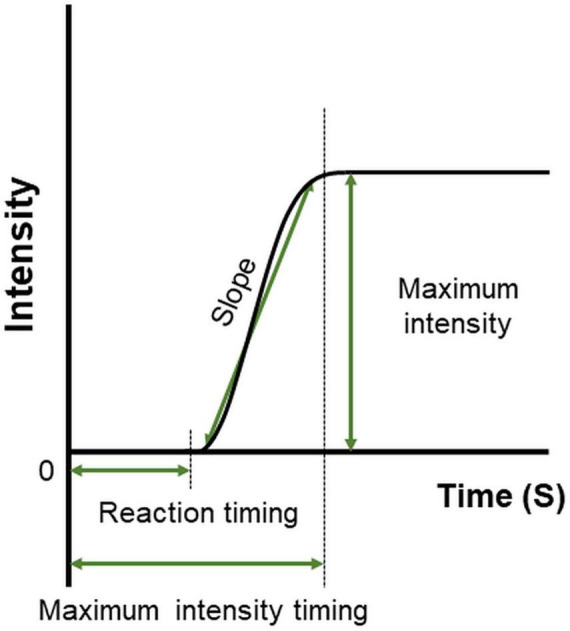
Features defined on the time–intensity profile. Reaction timing is the time at which the value of taste intensity starts to become larger than baseline intensity. The slope is the change in taste intensity per second. Maximum intensity is the highest and longest plateau on the profile. Maximum intensity timing is the time in milliseconds for the intensity curve to plateau.

The features of time–intensity profiles used for further analysis were as follows (Figure 4). (1) Maximum intensity (the mode of the smoothed intensity larger than 80% of the maximum smoothed intensity above the baseline; i.e., the highest and longest plateau on the profile), (2) reaction timing (the time at which the taste intensity value started to become larger than the baseline intensity), (3) maximum intensity timing (the time in milliseconds for the intensity curve to plateau), and (4) slope (the best-fit straight line was determined using linear regression from 10 selected time–intensity points) (40).

We used the Shapiro–Wilk test to test the normality of the distribution of the data. Nonparametric tests were then applied. We observed all data, and excluded strange values, which were the outliers identified through the statistical concept suggested by Tukey (42). Outliers for each participant were identified as those outside the following intervals: [Q1 − 1.5 IQR, Q3 + 1.5 IQR], where “Q” stands for “quartile” and “IQR” stands for “interquartile range” (42). We performed the Mann–Whitney U test to examine differences between older and young adult groups. Multiple comparisons were not performed.

## Results

### Experiment 1: cup tasting

**FIGURE 5:**
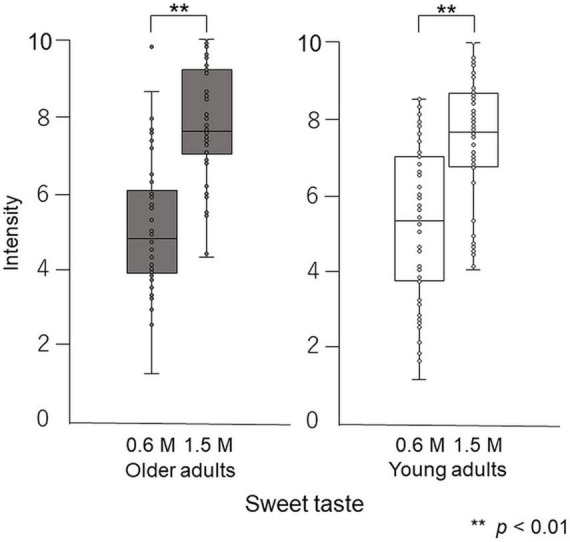
Differences in intensity between 0.6 M and 1.5 M glucose solutions in the cup tasting test (experiment 1). Both older and young adult participants perceived the taste intensity of 1.5 M glucose as significantly different from that of 0.6 M glucose (*p* < 0.01).

Paired-test results showed that participants perceived the taste intensity of 1.5 M glucose as significantly different from that of 0.6 M glucose (older adults *p* < 0.001, young adults *p* < 0.001). Participants perceived the sweet taste stimuli and significantly differentiated between the two concentrations (Figure 5).

**FIGURE 6:**
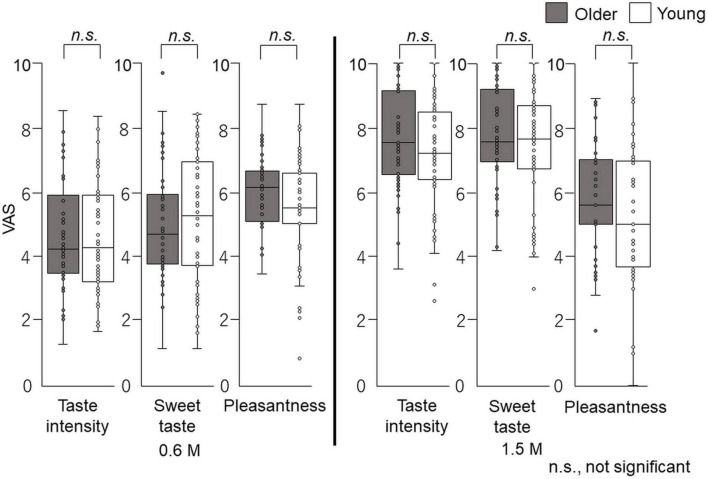
Questionnaire evaluation of the cup tasting test (experiment 1). Participants sipped the solution from a cup, held it on the tongue with no tongue movement, gargling, or swallowing, and spat it out after 6 s. The mouth was then washed with distilled water and static sweet taste intensity and pleasantness rated and recorded on a paper sheet. No significant differences were observed between older and young adults. Older adults, *n* = 40; young adults, *n* = 50.

The overall taste intensity, sweet intensity, and pleasantness in the time–intensity sensory evaluation are presented in Figure 6. No statistically significant differences between older adults and young adults were detected for taste intensity, sweet intensity, or pleasantness (Figure 6).

### Experiment 2: time–intensity sensory evaluation

**FIGURE 7:**
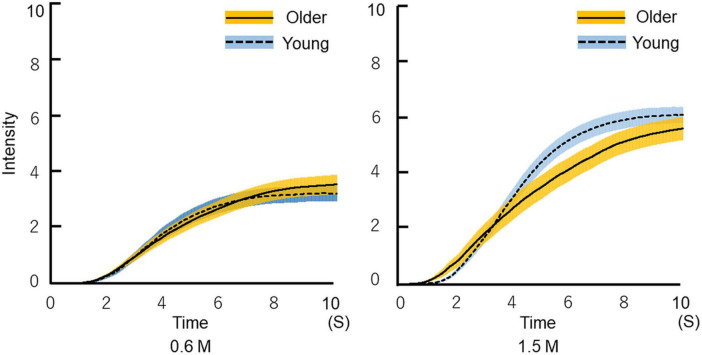
Time-intensity sensory evaluation of sweet taste intensity for all participants. The solutions were delivered to the tongue through a custom-made delivery system while participants recorded dynamic taste intensities on a hand-held time–intensity sensory evaluation meter. First, the 10 pairs of 0.6 M glucose for 10 s and distilled water for 10 s were delivered in a blocked design. Next, a 1.5 M solution was administered using the same method. We checked all time–intensity profiles. The profiles that included intensity meter operational errors were excluded. Then, the average of the replicated measurements of all profiles was calculated for each condition (the figure shows the mean ± SEM). The time required for older adults to begin to perceive sweetness was not different from that of young adults. Older adults perceived sweetness more slowly than young adults, and ultimately perceived almost the same intensity of sweetness as young adults. Older adults, *n* = 40; young adults, *n* = 50.

Time–intensity profiles are shown in Figure 7. The results indicate that older adults perceived sweet taste more slowly than young adults.

**FIGURE 8:**
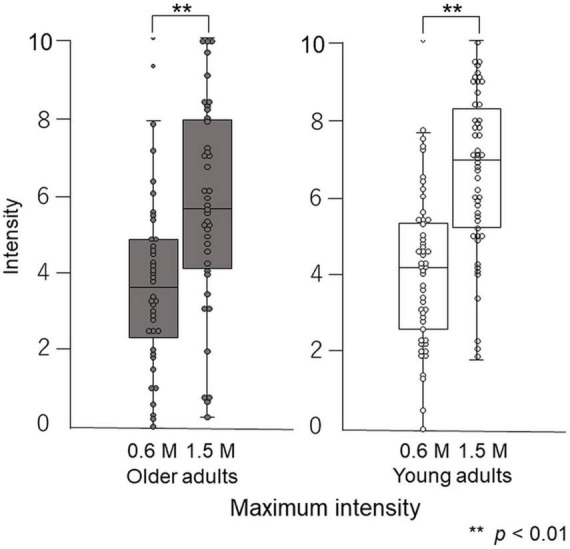
Differences in maximum intensity between 0.6 M and 1.5 M glucose solutions in time–intensity sensory evaluation. Both older and young adult participants perceived the taste intensity of 1.5 M glucose as significantly different from that of 0.6 M glucose (*p* < 0.01).

Paired-test results showed that participants perceived the taste intensity of 1.5 M glucose as significantly different from that of 0.6 M glucose (older adults *p* < 0.001, young adults *p* < 0.001). Participants perceived the sweet taste stimuli and significantly differentiated between the two concentrations (Figure 8).

### Maximum intensity

**FIGURE 9:**
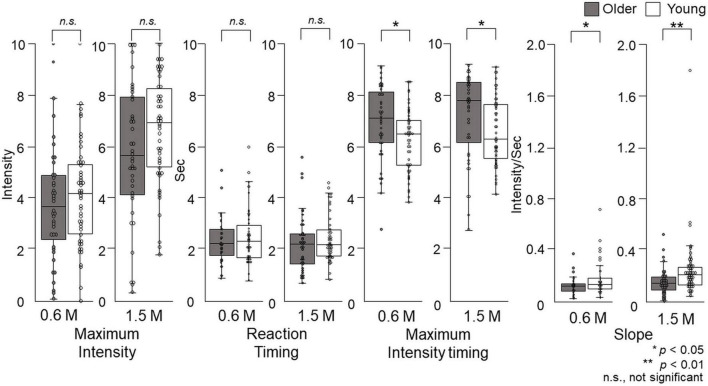
The features of the time–intensity profiles for the sweet taste of glucose. Maximum intensity, reaction timing, maximum intensity timing, and slope for the time–intensity sensory evaluation were used for further statistical analyses. Older adults did not exhibit significantly different maximum intensities or reaction timings for either concentration compared with young adults. The maximum intensity timings and slopes were significantly different between older adults and young adults for both concentrations. Older adults, *n* = 40; young adults, *n* = 50.

The maximum taste intensity of older adults was not significantly different from that of young adults (Figure 9).

### Reaction timing

Older adults did not significantly differ in their reaction timing compared with young adults (Figure 9).

### Maximum intensity timing

At both concentrations, there was a significant difference (*p* = 0.01) in the maximum intensity timing for older adults compared with young adults. For 0.6 M glucose, the median of maximum intensity timing was 7.1 (Q1 to Q3, 6.1–8.1) s for older adults and 6.4 (5.2–7.0) s for young adults. For 1.5 M glucose, the maximum intensity timing was 7.7 (6.1–8.4) s for older adults and 6.2 (5.5–7.6) s for young adults (*p* = 0.02) (Figure 9).

### Slope

At both concentrations, the slope of older adults was significantly different from that of young adults. For 0.6 M glucose, the slope of older adults was 0.10 (0.06–0.12) intensity per second (s), which was significantly different from the 0.11 (0.08–0.16) intensity/s for young adults (*p* = 0.04). For 1.5 M glucose, the slope of older adults was 0.14 (0.09–0.19) intensity/s, which was significantly different from the 0.21 (0.13–0.27) intensity/s of young adults (*p* = 0.003) (Figure 9).

### Questionnaire evaluation after sensory evaluation of time–intensity

**FIGURE 10:**
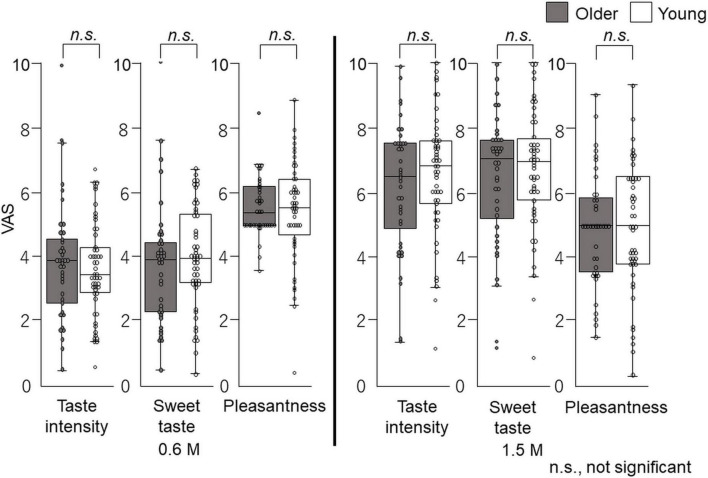
Questionnaire evaluation after time–intensity sensory evaluation (experiment 2). Immediately after the time–intensity sensory evaluation, participants reported the taste intensity, sweet taste intensity, and pleasantness perceived during the time–intensity sensory evaluation using the visual analog scale scores of the questionnaire. These results showed no significant differences in taste intensity, sweet taste intensity, or pleasantness between older and young adults for both concentrations. Older adults, *n* = 40; young adults, *n* = 50.

The overall taste intensity, sweet intensity, and pleasantness in the time–intensity sensory evaluation are presented in Figure 10. No statistically significant differences in taste intensity, sweet intensity, or pleasantness between older and young adults were seen.

## Discussion

In the cup tasting experiment, there were no statistically significant differences in VAS scores for taste intensity, sweet taste intensity, and pleasantness between young adults and older adults (Figure 6). Taste intensity findings have varied in previous reports. One reason for this is that the evaluation methods differ among studies (19–28). Therefore, to examine the differences between older and young adult groups, we used the intra-oral device and solution delivery system developed by Goto et al. (40) (Figure 2) and we obtained data under standardized conditions. Participants concentrated only on evaluating sweet taste intensity using an intensity meter and did not experience any stress. There are no published studies on time–intensity sensory evaluations of taste using a standardized delivery of taste solutions on the tongue for older adults.

Our results show that older adults were slower in perceiving changes in sweet intensity compared with young adults, while no significant differences were found for maximum intensities (Figures 7, 9). This may result from the aging of taste receptors and/or the central nervous system. In the present study, reaction timing is thought to reflect the time that elapses between a stimulus and action potential generation by taste receptors, and maximum intensity is the frequency of action potential generation in the central nervous system. The results of this study showed no significant difference in maximum intensity between older and young adults, and the significant difference in maximum intensity timing may result from the decline of nerve conduction velocity and synaptic delay.

A study examining the number of taste buds reported that infants have 240 taste buds per whole papilla, but that the number decreases with age (43). However, a study on the number of taste buds in human fungiform papillae reported no change with age, but large individual differences (44).

The taste receptors in taste buds are activated by sugars and sweeteners and regulate glucose transport. Sweet substances are recognized by TAS1R2 and TAS1R3 (45–48). The mRNA expression levels of TAS1R2 and TAS1R3 in the fungiform and circumvallate papillae are not significantly different between young and old mice (49). Furthermore, no significant effects of aging are seen for turnover rates of taste bud cells (49). Therefore, changes in taste sensitivity with aging are not caused by aging-related degradation of peripheral taste organs (49). Experiments in rats report no age differences in the electrophysiological responses of the chorda tympani nerve (50). Taste stimuli on the tongue generate signals that are transmitted to the brain via cranial nerves (51). Considering the results from previous studies, our results may also have an association with the central nervous system.

The time–intensity profile of sweet taste intensity of glucose in older adults has been characterized for the first time in this study. It is not possible to compare our results directly with previous studies. The results of taste intensity studies have varied in previous reports. One reason for this is that the evaluation methods differ among studies (29–36). However, using the same standardized system, we previously showed that for salty taste, older adults recognized taste intensity slowly and remained lower than that of young adults (37). However, in this study of sweet taste, only the slope and timing of maximum intensity differed significantly between the older and young adults, and there were no significant differences between older and young adults in reaction timing or maximum taste intensity (Figure 9). The reaction timing results show that older adults did not make slower decisions or physical responses during taste intensity evaluation. That is, older adults did not take long to perceive the sweet taste, but their recognition of taste intensity increased slowly. However, ultimately, their maximum taste intensity was not significantly different from that of young adults. Based on the results of this study, we recommend older adults “savor” to perceive sweet tastes at the same level experienced by young adults.

A limitation of this study is that the effect of medications taken by the older adults is not clear. In our literature survey, it was difficult to determine the incidence of drug-induced chemosensory disorders because functional measurements of chemosensory processes have not been performed in systematic well-controlled clinical trials (52). However, the incidence of adverse chemosensory effects from medications was 5% on average across most medications (52). In our study, 10 of the 40 older adults had taken medications for hyperlipidemia, reflux esophagitis, hypertension, and rheumatoid arthritis. The information for these medications list taste disorder as a side effect. All participants in the study distinguished between the two glucose solutions used, and the data distribution did not show any features associated with or without medication. Our data showed no significant differences in either maximum intensity or slope between older adults taking medications (10 participants) and those without medication (30 participants) (*p* > 0.05, Mann–Whitney U test). Adverse reaction from medication was not significant in our study; however, the effects of medication on taste should always be considered.

The investigators not being blind to the solution used represents a second potential limitation. We were aware of this limitation from our previous study of salty taste (37). Therefore, all authors checked all data and analyzed them repeatedly to avoid investigator bias (37). We included nearly all of the data, except for that affected by difficulties in using the intensity meter, as well as outliers identified through the statistical techniques suggested by Tukey (42).

A third potential limitation is that the participants did not receive complete sensory training. We appreciate that participant training can help reduce inter-individual variability in sensory evaluation and increase the reliability and reproducibility of the data. However, using the standardized system, participants concentrated only on the evaluation of sweet taste intensity and did not experience any stress. The participants remained motivated to participate in the series of experiments and participant bias due to fatigue was avoided.

The standardized system and thorough analysis at both personal and group levels allowed us to elicit physiological characteristics from the whole tongue. Participants perceived no tactile sense and no taste from taste receptors in the pharynx or gastrointestinal tract. The approach taken in this study was safe and can be used to inform future experimental studies with older adults. The risk of aspiration was very low using this solution delivery system because the participants did not need to swallow the solution. In fact, no participants in this study experienced aspiration.

The fourth potential limitation of this study is that we did not use general labeled magnitude scales (gLMS) but used VAS scores when participants rated pleasantness, sweet taste intensity, and taste intensity, in cup tasting and immediately after the time–intensity sensory evaluations (Figures 1, 6, 10). GLMS may be better for pleasantness but we employed the same method (VAS) as that used for the intensity assessments. We chose VAS for intensity considering the specific goals and context of our project, as suggested by Hayes et al. (53). Hayes et al. (53) showed that using sucrose samples ranging from 0.19 to 0.47 M, there was no clear advantage between gLMS and VAS because participants could differentiate the intensities of the sucrose samples. In addition, gLMS data show evidence of categorical behavior while VAS data do not. Participants exhibited substantial categorical behavior, clustering their responses near the verbal labels. Moreover, providing clear written instructions to rate between adjectives was not successful in reducing this behavior (53). Participants in this study focused on taste intensity only with no semantic information. Therefore, we assumed that VAS would be appropriate in this project.

Another potential limitation of the study is that no information about concentration of the sweet solution was provided to the participants. As a result, the intensity estimates in the cup tasting test (experiment 1) might have been higher than those recorded during and immediately after the time–intensity sensory evaluation (experiment 2). Participants’ comments and researchers’ observations indicated that the reason for the higher intensity estimates in the cup-sipping stimulation was mainly because of the participants’ first impression of sweetness. In the cup tasting test, participants tasted the 0.6 M solution for the first time. Many described it as “sweet” and gave it a high rating. During the time–intensity sensory evaluation, participants focused on perceiving the taste intensity. Other factors, such as repeated stimulations (10 times), longer stimulation time of a sweet taste, and sitting position vs. supine position, did not substantially affect participants’ evaluations.

## Conclusion

The time–intensity profile of sweet taste intensity of glucose in older adults has been characterized for the first time in this study. The time–intensity profile of older adults quantitatively showed that although the reaction timing required for older adults to begin perceiving sweetness was not different from that of young adults, they perceived sweetness more slowly than young adults, and ultimately perceived almost the same intensity of sweetness as young adults. These results provide a benchmark for sweet taste perception and useful dietary advice for the general public.

## Data availability statement

The raw data supporting the conclusions of this article will be made available by the authors, without undue reservation.

## Ethics statement

The studies involving humans were approved by the Institutional Review Board of Tokyo Dental College (No. 676). The studies were conducted in accordance with the local legislation and institutional requirements. The participants provided their written informed consent to participate in this study.

## Author contributions

HW: Conceptualization, Data curation, Formal analysis, Investigation, Methodology, Supervision, Validation, Visualization, Writing – original draft, Writing – review & editing. HM: Data curation, Investigation, Methodology, Validation, Writing – review & editing. MT: Data curation, Formal analysis, Methodology, Validation, Visualization, Writing – review & editing. HS: Data curation, Funding acquisition, Investigation, Methodology, Validation, Writing – review & editing. KI: Data curation, Investigation, Validation, Writing – review & editing. AI: Data curation, Investigation, Validation, Writing – review & editing. TG: Conceptualization, Data curation, Formal analysis, Funding acquisition, Investigation, Methodology, Supervision, Validation, Visualization, Writing – original draft, Writing – review & editing.

## Footnotes

1
www.r-project.org
